# Multiple aspects of lymphatic dysfunction in an *ApoE* ^*−/−*^ mouse model of hypercholesterolemia

**DOI:** 10.3389/fphys.2022.1098408

**Published:** 2023-01-06

**Authors:** Michael J. Davis, Joshua P. Scallan, Jorge A. Castorena-Gonzalez, Hae Jin Kim, Lim Hwee Ying, Yeo Kim Pin, Veronique Angeli

**Affiliations:** ^1^ Department of Medical Pharmacology and Physiology, University of Missouri, Columbia, MO, United States; ^2^ Department of Molecular Pharmacology, University of South Florida, Tampa, FL, United States; ^3^ Department of Pharmacology, Tulane University School of Medicine, New Orleans, LA, United States; ^4^ Immunology Translational Research Programme, Yong Loo Lin School of Medicine, Department of Microbiology and Immunology, National University of Singapore, Singapore, Singapore; ^5^ Immunology Programme, Life Sciences Institute, National University of Singapore, Singapore, Singapore

**Keywords:** lymphatic valve, permeability, contractile dysfunction, high fat diet, back-leak, pump limit, lymphatic muscle, lymphatic endothelium

## Abstract

**Introduction:** Rodent models of cardiovascular disease have uncovered various types of lymphatic vessel dysfunction that occur in association with atherosclerosis, type II diabetes and obesity. Previously, we presented *in vivo* evidence for impaired lymphatic drainage in apolipoprotein E null (*ApoE* ^*−/−*^) mice fed a high fat diet (HFD). Whether this impairment relates to the dysfunction of collecting lymphatics remains an open question. The *ApoE* ^*−/−*^ mouse is a well-established model of cardiovascular disease, in which a diet rich in fat and cholesterol on an *ApoE* deficient background accelerates the development of hypercholesteremia, atherosclerotic plaques and inflammation of the skin and other tissues. Here, we investigated various aspects of lymphatic function using *ex vivo* tests of collecting lymphatic vessels from *ApoE* ^*+/+*^ or *ApoE* ^*−/−*^ mice fed a HFD.

**Methods:** Popliteal collectors were excised from either strain and studied under defined conditions in which we could quantify changes in lymphatic contractile strength, lymph pump output, secondary valve function, and collecting vessel permeability.

**Results:** Our results show that all these aspects of lymphatic vessel function are altered in deleterious ways in this model of hypercholesterolemia.

**Discussion:** These findings extend previous *in vivo* observations suggesting significant dysfunction of lymphatic endothelial cells and smooth muscle cells from collecting vessels in association with a HFD on an *ApoE*-deficient background. An implication of our study is that collecting vessel dysfunction in this context may negatively impact the removal of cholesterol by the lymphatic system from the skin and the arterial wall and thereby exacerbate the progression and/or severity of atherosclerosis and associated inflammation.

## Introduction

Active lymphatic pumping is a major component of lymph transport. Active pumping is achieved by the spontaneous contractions of collecting lymphatics, which propel lymph centrally and act in concert with 1-way lymphatic valves, spaced at regular intervals, to prevent backflow (Scallan et al., 2016). Efficient lymph transport also depends on a low level of collecting lymphatic permeability, which normally is comparable to that of venules (Scallan & Huxley, 2010).

Rodent models of cardiovascular disease, including atherosclerosis, metabolic syndrome, obesity and advanced aging, have uncovered various types of lymphatic vessel dysfunction that occur in association with these diseases, as summarized in recent reviews (de Godoy, 2019; Jiang et al., 2019; Azhar et al., 2020; Ho & Srinivasan, 2020; Kataru et al., 2020; Norden & Kume, 2020). Lymphatic dysfunction may include a reduction in the frequency of spontaneous contractions, impaired contractile strength of lymphatic muscle cells (LMCs), increased permeability of the collecting vessel wall to water and solutes, and increased backflow through lymphatic valves, which normally are highly competent. Not all of the diseases listed above are characterized by every facet of lymphatic dysfunction, but many exhibit multiple components. Lymphatic dysfunction might not only be a consequence of these diseases, it could also contribute to their progression and/or manifestation. Precedent for this idea is provided by the observations by Rossitto et al. (Rossitto et al., 2020b) that even subtle amounts of edema can interfere with normal organ function (Rossitto et al., 2020a) and that subclinical edema with an underlying lymphatic component is present in patients and animal models of heart failure with preserved ejection fraction. Systemic subclinical lymphedema has also been noted in patients with a basal metabolic index (BMI) > 50 kg/m^2^ (de Godoy, 2019).

Previously, one of our laboratories provided *in vivo* evidence for lymphatic abnormalities in apolipoprotein E knockout (*ApoE* ^*−/−*^) mice fed a high fat diet (HFD) (Lim et al., 2009). This is a well-established mouse model of cardiovascular disease (Ilyas et al., 2022), in which a diet rich in fat and cholesterol on an *ApoE* deficient background accelerates the development of hypercholesteremia, atherosclerotic plaque development (Zhao et al., 2020) and inflammation of the skin and other tissues (Feingold et al., 1995; van Ree et al., 1995). Lim et al. (Lim et al., 2009) showed that ApoE^−/−^ mice fed a diet rich in fat and cholesterol exhibited evidence of swelling in the tail and foot pads, pointing to possible disruption in lymphatic drainage. Histological examination revealed fluid pockets and increased lipid deposition in the dermis as well as increased infiltration of CD45^+^ leukocytes. Collecting lymphatic vessels in the ear skin of these mice exhibited evidence of hyperpermeability, based on the extravasation and/or reflux of Evans Blue dye after uptake from the interstitium. Two subsequent studies showed that the transport of macromolecules, such as fluorescein isothiocyanate dextran, in the skin (Lim et al., 2013) and aortic lymphatic vessels (Yeo et al., 2020) was significantly reduced in *ApoE* ^*−/−*^ mice compared to *ApoE* ^*+/+*^ mice. Collecting vessels in skin also failed to downregulate Lyve1 upon maturation and showed reduced smooth muscle cell coverage, compared to *ApoE* ^*+/+*^ controls (Lim et al., 2009). Collectively, these findings were suggestive of altered collecting vessel function.

In the present study, we directly investigated various aspects of lymphatic collecting vessel function in ApoE-deficient mice on a HFD. Vessels were excised from *ApoE* ^*+/+*^ or *ApoE* ^*−/−*^ mice fed a HFD and studied under defined conditions *ex vivo*, in which we could quantify possible changes in lymphatic contractile strength, spontaneous contraction frequency, calculated and measured lymph pump output, secondary valve function, and collecting vessel permeability to albumin. Our results show that all of these aspects of lymphatic vessel function are compromised in *ApoE* ^*−/−*^ mice, compared to *ApoE* ^*+/+*^ control mice, fed a HFD. These findings extend those of other groups (Blum et al., 2014; Garcia Nores et al., 2016; Nitti et al., 2016; Castorena-Gonzalez, 2022) suggesting that significant dysfunction of lymphatic collecting vessels develops in association with a high fat diet, and extend our previous *in vivo* observations (Lim et al., 2009; Lim et al., 2013; Yeo et al., 2020), showing that lymphatic dysfunction is further exacerbated by *ApoE* deficiency. The combination of *ApoE* deficiency and HFD negatively impacts both lymphatic smooth muscle (LMC) and lymphatic endothelial cell (LEC) function, which may result in impaired removal of cholesterol by the lymphatic system from the skin and the arterial wall (Lim et al., 2013; Martel et al., 2013; Martel & Randolph, 2013) and thereby exacerbate the progression and/or severity of atherosclerosis (Yeo KP et al., 2020).

## Methods

### Mice

All procedures were approved by the animal care committees at the University of Missouri and National University of Singapore and complied with the standards stated in the “Guide for the Care and Use of Laboratory Animals” (National Institutes of Health, revised 2011). *ApoE* ^*−/−*^ and *ApoE* ^*+/+*^ mice on the C57BL/6 background were obtained from Jackson laboratory (Bar Harbor, ME). Male and female mice were fed a diet rich in fat and cholesterol (21% milk fat and .15% cholesterol, Harlan Teklad) from 6 weeks of age for 14–16 weeks, as described in our previous studies (Lim et al., 2009; Lim et al., 2013; Yeo et al., 2020). *ApoE* ^*−/−*^ mice of both sexes fed a HFD developed hypercholesterolemia and atherosclerosis (Lim et al., 2009). *ApoE* ^*+/+*^ mice had average weights of 28.2 ± 4.9 (SD) g, whereas *ApoE* ^*−/−*^ mice had average weights of 24.1 ± 1.5 (SD) g; these values were significantly different (Mann-Whitney test, *p* = .0225). Similar weight differences between these two strains have been reported in other studies (Schreyer et al., 2002; Chiba et al., 2003; Hofmann et al., 2008).

### Vessel isolation and cannulation

20–22 week old mice were anesthetized with sodium pentobarbital (100 mg/kg, i. p.) and placed face down on a heated pad. Popliteal lymphatic vessels were exposed through a superficial incision in the back of the leg, removed and transferred to a dissection chamber filled with Krebs-albumin solution for further dissection. For mesenteric vessel isolation, the abdomen was opened and the entire small intestine was removed and pinned out, after which lymphatic collectors were excised from the duodenum. Individual vessels containing three to four valves were pinned to the base of a Sylgard chamber and partially cleaned of fat and connective tissue. A 1-valve vessel was then transferred to a 3-ml myograph chamber containing Krebs-albumin solution, cannulated at each end with a glass micropipette (40–50 μm OD), pressurized to 3 cmH_2_O, and further cleaned. The chamber, with associated micropipettes, pipette holders and micromanipulators, was transferred to the stage of an inverted microscope. The same pair of cannulation pipettes was used for all experiments in order to standardize valve function measurements. Polyethylene tubing connected the back of each micropipette to a low pressure transducer and analog pressure controller (Scallan et al., 2013), allowing independent control of inflow (Pin) and outflow (Pout) pressures. Custom LabVIEW programs (National Instruments; Austin, TX) acquired analog data from the pressure transducers at 30–40 fps simultaneously with vessel inner diameter, as detected from video images acquired using a Basler firewire camera (Davis, 2005). Digital videos of the valve function protocols, with embedded pressure data, were recorded for off-line analyses. To minimize axial bowing of the vessel at higher intraluminal pressures, Pin and Pout were briefly set to 10 cmH_2_O at the beginning of every experiment and the segment was stretched axially to remove slack. After the pressures were returned to 3 cmH_2_O, the vessel was allowed to equilibrate in Krebs buffer at 37°C for at least 20 min until spontaneous contractions developed and the contraction pattern stabilized. Constant exchange of buffer was maintained using a peristaltic pump at a rate of .5 ml/min.

### Valve function tests

After a multi-valve segment was set up on the microscope, the segment was shortened to a single valve for valve function tests and the rest of the vessel was stored at room temperature for later re-cannulation. Valve tests were conducted in Ca^2+^-free Krebs buffer at 37°C to eliminate spontaneous contractions that otherwise would have interfered with the tests. Luminal pressure on the inflow side of the valve (Psn) was measured with a servo-null micropipette inserted through the wall. A sharply-tapered pilot micropipette was used to make an initial hole in the vessel wall upstream from the valve. That pipette was then removed and replaced with a less-tapered servo-null micropipette with a tip diameter of 3–5 μm. After insertion, the servo-null micropipette was advanced to seal the hole. The calibration of the servo-null pipette was checked, and adjusted as needed, after raising Pin and Pout simultaneously between .5 and 10 cmH_2_O and changing the gain/offset of the Psn amplifier signal.

To ensure accurate and consistent measurements of valve back-leak, 1) all three transducers (Pin, Psn, Pout) were calibrated before each experiment; 2) the pipettes were cleaned after each experiment and checked before and during each valve test to ensure that the tips were free of debris; 3) the lines were free of bubbles; 4) the Psn pipette calibration was rechecked at the end of the valve test.

The first test of valve function measured the back-leak of pressure through a closed valve. Starting with Pin and Pout = .5 cmH_2_O, and the valve open, Pout was raised, ramp-wise, to 10 cmH_2_O over ∼1 min period while Pin was held at .5 cmH_2_O. A normal valve closed as Pout exceeded ∼1 cmH_2_O and remained closed for the duration of the Pout ramp. Pressure back-leak through the closed valve was measured on the inflow side of the vessel using the servo-null micropipette, which could resolve changes as small as ∼.05 cmH_2_O. The value of Psn at Pout = 10 cmH_2_O was sometimes used as a representative index of back-leak, but additional values of Psn at intermediate Pout levels were determined offline using a LabView program after binning the Psn data in .5 cmH_2_O Pout intervals.

The second test determined the adverse pressure gradient (ΔP, Pout—Pin) required to close an initially open valve. Because this value increases with increasing vessel diameter (Davis et al., 2011; Lapinski et al., 2017), the measurements were made over a wide range of baseline pressures, each of which in turn determined the baseline diameter. Starting with the valve open, output pressure was raised ramp-wise and the ΔP was determined at the instant of valve closure. The test was repeated for baseline pressures .1, .2, .3, .5, 1, 2, 3, 5, 8, 10 cmH_2_O, which resulted in measurements over a range of diameters spanning ∼40%–100% of the maximal passive diameter. ΔP for closure was then plotted against Pin or normalized diameter. The highest ΔP that we could test was 30 cmH_2_O (equating to a maximum Pout of 40 cmH_2_O when Pin was 10 cmH_2_O) without exceeding the specified safety range the ultra-sensitive pressure sensor elements.

### Pump tests

To assess the pumping ability of single, 2-valve lymphangions, we conducted an additional *ex vivo* contraction protocol, similar to that described previously (Davis et al., 2012b; Lapinski et al., 2017; Castorena-Gonzalez et al., 2018). With Pin and Pout set at .5, 1 or 2 cmH_2_O (some vessels had no contractions at .5 or 1 cmH_2_O), Pout was elevated ramp-wise, usually to 10 cmH_2_O, at a rate of ∼3 cmH_2_O/min, with Pin held constant, while monitoring the position of the outflow valve. Successful ejection during a contraction cycle was associated with opening of the outflow valve when the peak of the contraction-induced internal pressure spike transiently exceeded Pout (Davis et al., 2011); if the peak did not exceed Pout, the outflow valve never opened during lymphatic systole. As Pout was elevated, the pump eventually weakened such that the Pout value at the time of the last successful ejection corresponded to the “pump limit” of the lymphangion. Close agreement between the output valve position and the peak of the systolic internal pressure spike (as measured between the valves using a servo-nulling micropipette) was demonstrated previously (Davis et al., 2012a; Lapinski et al., 2017; Castorena-Gonzalez et al., 2018), so that it was not necessary to measure Psn in every experiment. Valve position, either open or closed, was determined from replay of the recorded protocol videos using a custom LabVIEW program, as described previously (Davis et al., 2012b), while maintaining synchronization of the valve position data with the pressure and diameter data.

### Measurement of lymphatic vessel solute permeability

Albumin flux (J_s_, mmol/s) across the lymphatic wall was measured directly using microscope-based photometry on a Zeiss Axiovert microscope equipped with appropriate optics and filters for measuring FITC fluorescence. A mesenteric collecting lymphatic was excised and cannulated as described above. To control perfusion and pressure, each micropipette was connected to manual water manometers *via* polyethylene tubing. Two manometers were connected through a valve to each side of the dual-lumen perfusion micropipette, such that when the valve was turned, one side of the pipette or the other selectively perfused the lymphatic vessel without changing intraluminal hydrostatic pressure. This allowed controlled perfusion of either the unlabeled or fluorescent BSA. A fraction of the perfused albumin was labeled with a fluorescent dye (Alexa-488) and fluorescence intensity was measured within a rectangular region of interest defined by an adjustable window in front of a photometer (PTI 814, Photonic Technology International) that sampled light from the vessel lumen and adjacent extravascular space. The photometer output was digitized using an analog-to-digital converter (National Instruments) and analyzed off-line. Based on pilot experiments, the vessels were pressurized to 9 cmH_2_O on the inlet side and 5 cmH_2_O on the outflow side to enable rapid changes in the perfusate. To make a measurement, unlabeled BSA was perfused first to obtain the background fluorescence intensity. Switching to perfusion of the fluorescent BSA caused a rapid, step-increase in fluorescence intensity (I_o_) on the photometer. Over time, the fluorescent BSA moved across the lymphatic vessel wall into the bath solution, which caused a gradual but linear increase in photometer voltage (dI_f_/dt). Changing the perfusate to the unlabeled BSA washed away all fluorescence, eventually returning the photometer signal to baseline, and allowed repeated measurements to be made. Albumin permeability (P_s_, cm/s) was then calculated from a rederived form of Fick’s first law relating albumin flux to a constant surface area (S, cm^2^) and concentration gradient (∆C, mmol/mL):

$$\left.P_{s}{=J}_{s}/S\Delta {C=(1/I}_{o}{)(dI}_{f}/dt)(D/4\right)$$ (1)

In which collecting lymphatic diameter (D,cm) was simultaneously tracked on a transmitted-light, near infrared image of the vessel displayed on a computer monitor to ensure that it did not change throughout each recording. There was no overlap between the near infrared light and the fluorescence emission of Alexa-488-BSA.

### Immunostaining of popliteal collectors

Dissected popliteal collecting lymphatics were fixed with 2% paraformaldehyde and blocked with phosphate-buffered saline (PBS) containing .3% Triton X-100% and .5% bovine serum albumin. Samples were incubated with primary FITC-conjugated monoclonal anti-smooth muscle actin (Sigma-Aldrich). Samples were mounted on a slide with Dako fluorescent mounting medium and coverslip and then viewed with a confocal microscope (Leica TCS SP5, Leica Microsystems Inc., Deerfield, IL) with LAS AF confocal software (version 1.8.2, Leica Microsystems Inc.).

### Solutions and chemicals

Krebs buffer contained: 146.9 mM NaCl, 4.7 mM KCl, 2 mM CaCl_2_·2H_2_O, 1.2 mM MgSO_4_, 1.2 mM NaH_2_PO_4_·H_2_O, 3 mM NaHCO_3_, 1.5 mM Na-HEPES, and 5 mM d-glucose (pH = 7.4). An identical buffer (“Krebs-BSA”) also contained .5% bovine serum albumin. Krebs-BSA buffer was present both luminally and abluminally during cannulation, but the abluminal solution was constantly exchanged with plain Krebs during the experimental protocol. For Ca^2+^-free Krebs, 3 mM EGTA replaced CaCl_2_·2H_2_O. All chemicals were obtained from Sigma-Aldrich (St. Louis, MO) except BSA (US Biochemicals; Cleveland, OH), MgSO_4_ and Na-HEPES (ThermoFisher Scientific; Pittsburgh, PA).

### Statistical tests

The number *n* refers to the number of vessels included per group. There were no obvious differences in lymphatic function between male and female mice in our protocols so data from both sexes were combined for statistical analyses. The normality of data sets was assessed using Kolmogorov-Smirnov tests. Statistical differences between normally distributed data sets were assessed *via* mixed-effects model ANOVAs with Geisser-Greenhouse correction (allowing for missing values or unequal groups) with Fisher’s or Sidak’s multiple comparisons tests, as specified in the individual figure legends. Mann-Whitney tests were used to compare differences in groups that were not normally distributed. Statistical analyses were performed using Prism9 (Graphpad). Data are plotted as mean ± SEM. Unless otherwise stated, the significance level was *p* < .05.

## Results

**FIGURE 1:**
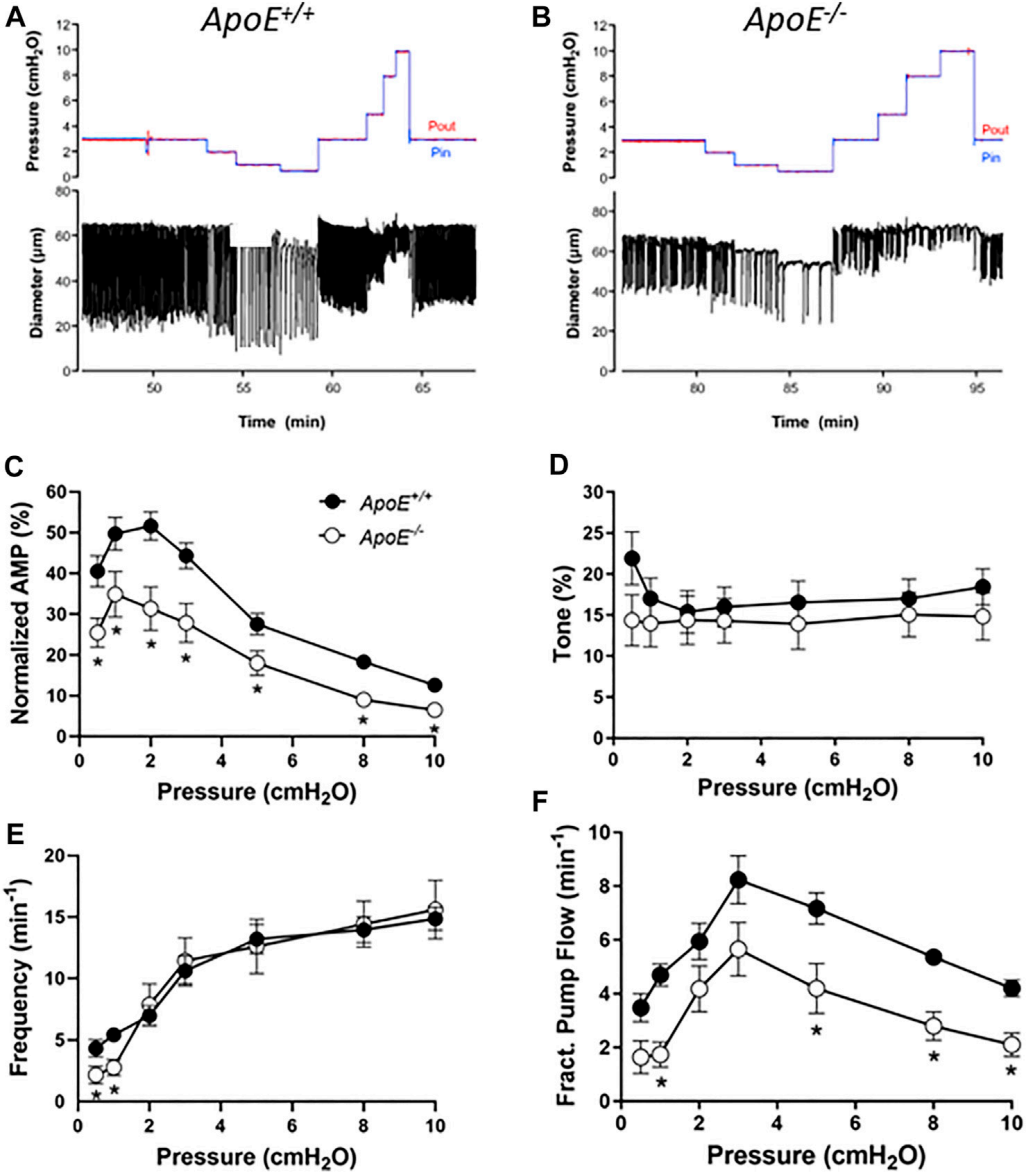
Contractile parameters of *ApoE* ^*+/+*^ and *ApoE* ^*−/−*^ popliteal lymphatic vessels. Contractile strength was consistently reduced at all pressures between .5 and 10 cmH_2_0 in *ApoE* ^*−/−*^ vessels. **(A)** Recording of spontaneous contractions from an *ApoE* ^*+/+*^ popliteal lymphatic vessel at different levels of luminal pressure. Each downward deflection in diameter represents a single, twitch contraction. **(B)** Recording of spontaneous contractions from an *ApoE* ^*−/−*^ lymphatic in response to the same protocol. Note the relatively reduced contraction amplitude at all pressures and reduced contraction frequency at the lowest pressure. **(C)** Summary data for normalized amplitude; changes in ejection fraction (not shown) were similar. **(D)** Summary data for tone, which was significantly different at only the lowest pressure (the least reliable measurement of tone). **(E)** Summary data for frequency, which was significantly different at the two lowest pressures. **(F)** Fractional pump flow was significantly reduced in *ApoE* ^*−/−*^ vessels at about half the pressures. Statistical differences were assessed *via* mixed-effects model ANOVAs with Geisser-Greenhouse correction (allowing for missing values or unequal groups) with Fisher’s LSD multiple comparisons *post hoc* tests.

Popliteal collectors from *ApoE* ^*−/−*^ mice typically exhibited blunted contraction amplitudes compared to *ApoE* ^*+/+*^ vessels (with both strains fed a HFD). Representative examples of spontaneous contractions as a function of pressure are shown in Figures 1A,B for the two strains. Smaller contraction amplitudes are apparent for the *ApoE* ^*−/−*^ vessel at all pressures studied as well as a lower frequency at pressure = .5 cmH_2_O (Figure 1B). Because the absolute amplitude is in part determined by vessel size, which can vary between animals and strains, the summary data in panel C are expressed as amplitude normalized to the maximum passive diameter. This analysis confirms that the normalized amplitudes of *ApoE* ^*−/−*^ vessels were ∼60% of that for *ApoE* ^*+/+*^ vessels, and these differences were statistically significant at all pressures between .5 and 10 cmH_2_O. In contrast, there were no significant differences in tone between *ApoE* ^*+/+*^ and *ApoE* ^*−/−*^ vessels at any pressure (Figure 1D). *ApoE* ^*−/−*^ vessels had a significantly lower frequency of spontaneous contractions at each the two lowest pressures, .5 and 1 cmH_2_O, but comparable frequencies to *ApoE* ^*+/+*^ vessels at all other pressures (Figure 1E). Calculated flow (FPF) for *ApoE* ^*−/−*^ vessels was 60%–80% of that for *ApoE* ^*+/+*^ vessels at all pressures, with the differences being significant at about half those pressures (Figure 1F). It should be noted that one vessel was excluded from this analysis because it appeared to be an outlier: 2 of 9 *ApoE* ^*−/−*^ vessels had no contractions at either of the two lowest pressures and 4 of 9 *ApoE* ^*−/−*^ vessels had contraction frequencies <.5 per min at the two lowest pressures, but 1 vessel had a 3x higher frequency and 3x higher tone than the average for *ApoE* ^*+/+*^ vessels; for that reason it was considered an outlier. Inclusion of that vessel eliminated any statistical differences between *ApoE* ^*+/+*^ and *ApoE* ^*−/−*^ vessels for tone or frequency but did not change the differences for normalized amplitude or FPF. These results suggest an impairment in contractile function at all pressures in *ApoE* ^*−/−*^ vessels and impairment in the pacemaking mechanism at the two lowest pressures. The failure of the *ApoE* ^*−/−*^ vessels to produce spontaneous contractions at pressures <2 cmH_2_O is a pattern that we have observed in other models of contractile dysfunction (Zawieja et al., 2019; Davis et al., 2020).

**FIGURE 2:**
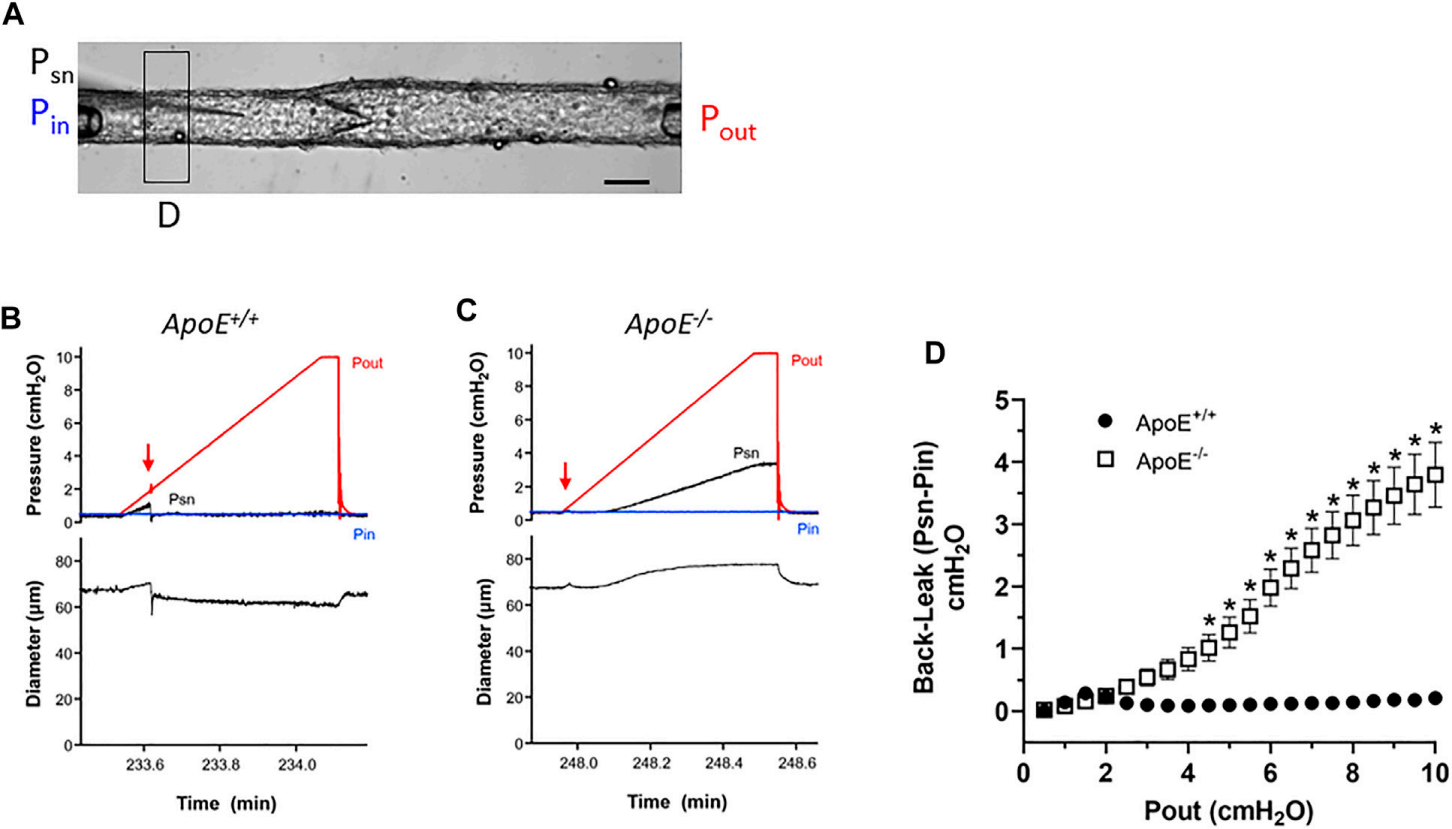
*ApoE* ^*−/−*^ valves exhibit increased back-leak. **(A)** Experimental configuration used for valve tests. Calibration bar = 40 μm. **(B–C)** Examples of back-leak tests over the pressure range 0–10 cmH_2_O for isolated valves from *ApoE* ^*+/+*^ and *ApoE* ^*−/−*^ popliteal lymphatics. When closed, the *ApoE* ^*+/+*^ valve showed only very slight back leak **(B)**, comparable to that of normal valves in previous studies; **(C)** in contrast, the *ApoE* ^*−/−*^ valve began to leak when Pout exceeded ∼3 cmH_2_O. **(D)** Summary data show that *ApoE* ^*+/+*^ valves, when closed, effectively prevent back-leak whereas *ApoE* ^*−/−*^ valves consistently showed back-leak when Pout exceeded ∼3 cmH_2_O; back-leak was significant at all Pout levels ≥4 cmH_2_O. Statistical differences were assessed *via* mixed-effects model ANOVAs with Geisser-Greenhouse correction with Sidak’s multiple comparisons tests.

Next, we conducted tests of lymphatic valve function using methods described previously (Davis et al., 2011; Sabine et al., 2018). For these tests, collecting segments containing only a single valve were studied in Ca^2+^-free Krebs solution to eliminate spontaneous contractions that would interfere with the tests. A diagram illustrating the experimental set-up is provided in Figure 2A. During cannulation and cleaning, the vessel segment was rotated so that the valve leaflets were oriented as in this image. A representative recording of a back-leak test for an *ApoE* ^*+/+*^ vessel is shown in Figure 2B. Initially, with Pin = Pout = .5 cmH_2_O, the valve was open and the local pressure upstream from the valve measured with a servo-null micropipette (Psn) rose as the Pout ramp began until Pout reached ∼1.8 cmH_2_O when the valve snapped closed (arrow). After that time Psn stayed at a low level even as Pout reached 10 cmH_2_O. The *ApoE* ^*−/−*^ valve in Figure 2C also closed almost immediately after the start of the Pout ramp (arrow) but once Pout reached ∼3 cmH_2_O, Psn began to increase until it reached a value of 3.5 cmH_2_O at Pout = 10 cmH_2_O. The concomitant rise in D upstream from the valve during the Pout ramp confirms that the *ApoE* ^*−/−*^ valve was leaky. The pressure back-leak (Psn-Pin) at Pout = 10 cmH_2_O was 0 cmH_2_O for the *ApoE* ^*+/+*^ valve and 3.0 for the *ApoE* ^*−/−*^ valve.

Summary data sets for the two genotypes are shown in Figure 2D. To analyze the changes in Psn during Pout ramps, the Psn recordings were aligned, binned in .5 cmH_2_O Pout intervals and an average value ±SEM for each bin was obtained using a LabView program. At Pout = 10 cmH_2_O, *ApoE* ^*+/+*^ valves showed only very slight back-leak (.1 cmH_2_O), but back-leak was much more severe for *ApoE* ^*−/−*^ valves and the differences were statistically significant at Pout levels ≥4 cmH_2_O.

**FIGURE 3:**
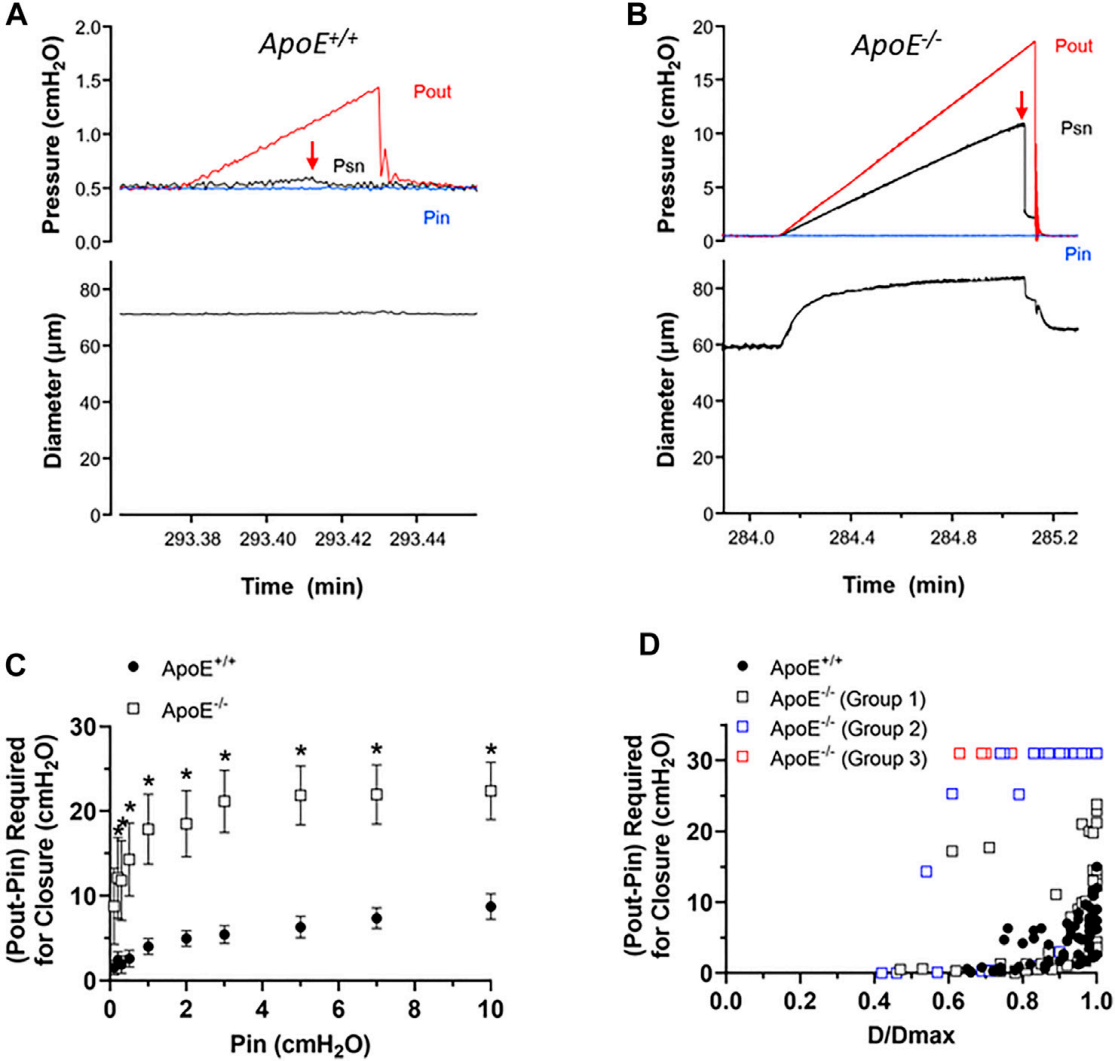
*ApoE* ^*−/−*^ valves require a higher adverse pressure gradient for closure. **(A–B)** Examples of valve closure tests for isolated *ApoE* ^*+/+*^ **(A)** and *ApoE* ^*−/−*^ **(B)** valves. Note the difference in the *y*-axis scales between the two panels. **(C)** Summary data showing that normal valves require a slightly higher adverse (Pout-Pin) pressure gradient (ΔP) to close a valve as baseline pressure (Pin) rises. **(D)** When ΔP for closure is plotted as a function of normalized diameter, the relationship is curvilinear. A gradient of .1 cmH_2_O is sufficient to close a typical valve at low diameter (∼.6 D/Dmax), whereas a higher pressure gradient (2–15 cmH_2_O) is needed near maximal diameter. 7 of 7 *ApoE* ^*+/+*^ valves closed within the normal range of adverse pressure gradients. In contrast, 5 of 11 *ApoE* ^*−/−*^ valves showed normal closure curves, 4 of 11 closed at low diameters but would not close at higher diameters, and 2 of 11 *ApoE* ^*−/−*^ valves would not close at any adverse pressure gradient tested. Statistical differences were assessed *via* mixed-effects model ANOVAs with Geisser-Greenhouse correction with Sidak’s multiple comparisons tests.

Next, we tested for possible changes in the ability of valve leaflets to close at fixed levels of an adverse pressure gradient. The experimental set-up was the same as shown in Figure 2. Representative recordings of valve closure tests are shown in Figures 3A,B. With Pin held at .5 cmH_2_O, the *ApoE* ^*+/+*^ valve closed when the Pout ramp reached ∼1.2 cmH_2_O (Figure 3A, arrow). Under similar conditions, the *ApoE* ^*−/−*^ valve did not close until Pout reached ∼18 cmH_2_O (Figure 3B, arrow).

Summary data for the valve tests are shown in Figures 3C,D. The relationship between the ΔP for closure (i.e. the difference between Pout–Pin at the point of valve closure) and Pin, which was used to set the baseline diameter for the test, is shown in Figure 3C. ΔP for closure values were significantly higher for *ApoE* ^*−/−*^ valves at all pressures except the lowest one. The individual values of ΔP for closure for each valve as a function of normalized diameter are shown in Figure 3D, and are described by a curvilinear function as for other genotypes (Davis et al., 2011). *ApoE* ^*+/+*^ valves closed at ΔP < 1 cmH_2_O when D values were ∼70% of Dmax and required somewhat higher pressures in the range of 2–15 cmH_2_O when D was maximal (set by increasing Pin to 10 cmH_2_O). The value of ΔP at Dmax is somewhat higher than for other “control” mouse genotypes fed a normal diet (Sabine et al., 2015; Lapinski et al., 2017; Chen et al., 2020; Scallan et al., 2021) and it is not clear if this reflects the influence of the HFD *per se*. The behavior of about half the *ApoE* ^*−/−*^ valves was similar in that 5 of 11 *ApoE* ^*−/−*^ valves showed a relatively low ΔP for closure even at Dmax. However, 4 of 11 *ApoE* ^*−/−*^ valves closed at low D values but never closed at higher D values and 2 of 11 *ApoE* ^*−/−*^ valves never closed at any diameter tested, even under an imposed adverse ΔP = 30 cmH_2_O. As explained in previous publications, we propose that an upward shift in the ΔP for closure vs. D/Dmax relationship reflects an increase in valve leaflet stiffness. Thus, the 3 different types of behavior for the *ApoE* ^*−/−*^ valves likely represent one population of ∼normal valves, one group with increased stiffness that retards closure under physiological conditions, and another group that is intermediate, possibly in transition from normal to increased stiffness.

Because FPF is only an estimate of the pumping ability of a collecting lymphatic vessel and is typically determined under conditions when Pin and Pout are equal, we performed additional tests to directly assess the ability of 2-valve lymphangions to pump against an adverse pressure gradient. Such gradients are generated in lymphatic networks by the intrinsic pumping of each branching order of collectors and are exacerbated by the imposition of gravitational loads (Olszewski, 1977; Olszewski & Engeset, 1980). Indeed, this is the reason why human lymphedema most often occurs in dependent extremities.

**FIGURE 4:**
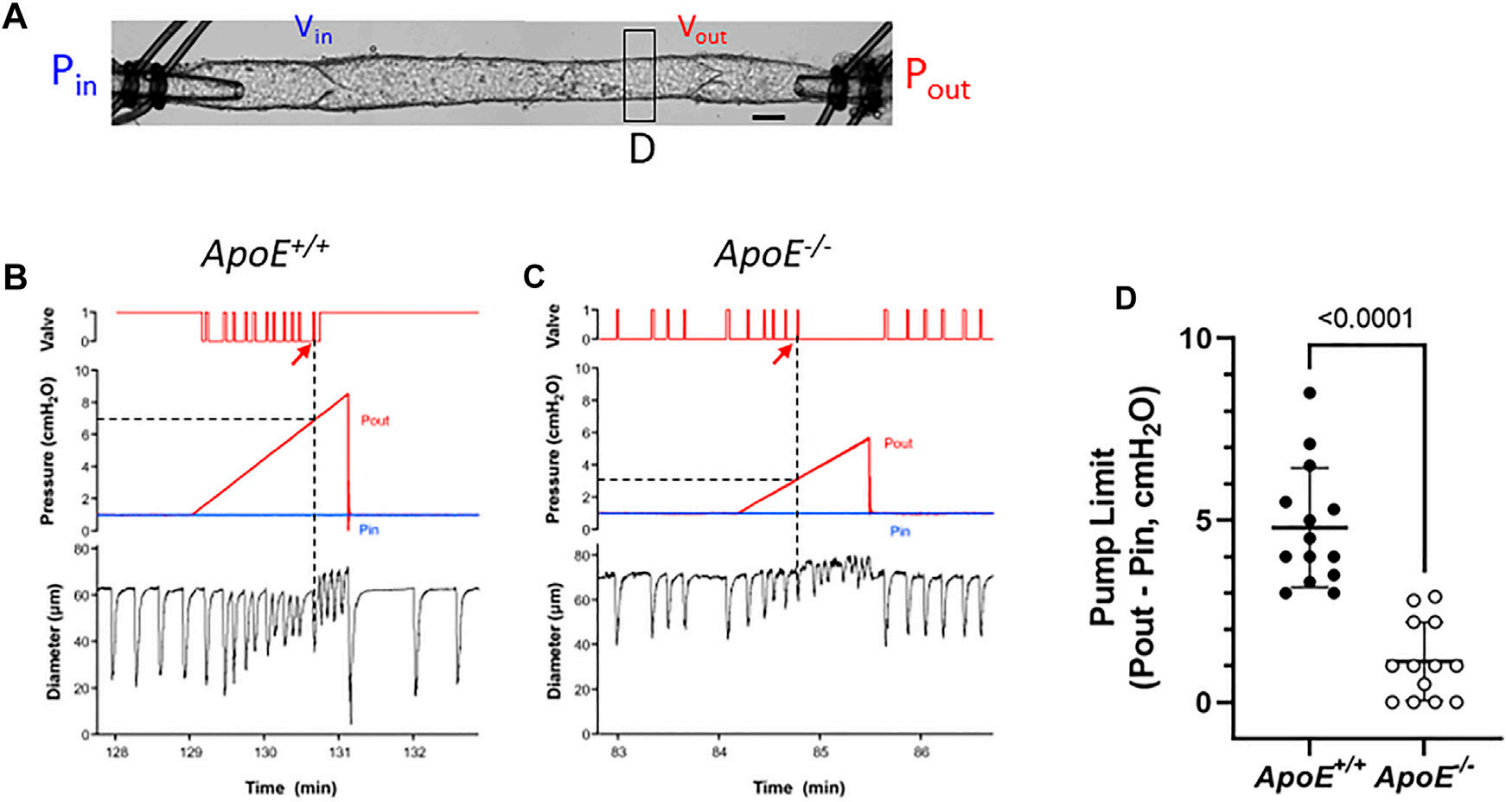
*ApoE* ^*−/−*^ vessels have impaired pump strength. **(A)** Experimental configuration used for pump test of a 2-valve lymphangion. Calibration bar = 35 μm. Pump test examples for an *ApoE* ^*+/+*^ vessel **(B)** and a *ApoE* ^*−/−*^ vessel **(C)**. **(D)** Pump test results for isolated, 2-valve popliteal lymphatic segments from *ApoE* ^*+/+*^ and *ApoE* ^*−/−*^ mice. On average *ApoE* ^*−/−*^ vessels were only able to pump against an adverse pressure gradient of 1.2 cmH_2_O, compared to 4.8 cmH_2_O for *ApoE* ^*+/+*^ vessels. Statistical differences were assessed *via* Mann-Whitney test. Valve positions: 1 = open; 0 = closed.

For pump tests, a 2-valve segment (i.e., a complete lymphangion) was used. Pin was held constant at a low value while Pout (which is equivalent to afterload under these conditions) was raised ramp-wise (typically to 8–10 cmH_2_O), as shown in Figure 4A. Diameter was measured on the upstream side of the outflow valve and playback of the recorded video was used to determine the outflow valve position (either open or closed) in each video frame. Once the Pout ramp began, the outflow valve was closed except for a brief period of time at the peak of each lymphatic systole when the valve transiently opened [as the internal pressure spike exceeded Pout (Davis et al., 2011)]. As Pout continued to rise, the ability of the pump to eject its contents was exceeded at some point, as evident by the failure of the output valve to open during a contraction. The pump limit, P_limit_, was defined as the value of Pout (minus Pin) at the time of last successful ejection. In the representative examples shown in Figure 4, P_limit_ for *ApoE* ^*+/+*^ vessel (panel A) was 6 cmH_2_O (after subtraction of Pin) and P_limit_ for the *ApoE* ^*−/−*^ vessel was 2.2 cmH_2_O (panel B). The pump test data are summarized in Figure 4D and show that popliteal collectors from *ApoE* ^*−/−*^ mice have a significantly lower pump strength (1.2 vs. 4.8 cmH_2_O) than comparable vessels from *ApoE* ^*+/+*^ mice, in agreement with the lower calculated pump flows for *ApoE* ^*−/−*^ vessels in Figure 1F.

A complicating factor in this protocol was the tendency for the outflow valve to sometimes “lock” into the open position during the Pout ramp; it can be forced into this position if the inflow valve ceases to open in diastole. This phenomenon is described in a previous publication (Bertram et al., 2017) and occurs if the respective local adverse pressure gradients across the two valves favor closure of the inflow valve rather than the outflow valve. In such cases, we determined P_limit_ from the previous (successful) ejection prior to outflow valve “lock”. This likely underestimated the actual P_limit_, but a minimal estimate of P_limit_ could at least be made. An example of this behavior is shown in Figure 4A (on the contraction after the arrow). As shown in this case, output valve “lock” was also accompanied by a sudden increase in D (coinciding with the vertical line in panel A); prior to that time D declined slightly with rising Pout (Davis et al., 2009; Davis et al., 2012b). Valve “lock” did not occur in the *ApoE* ^*−/−*^ vessel; in that case D increased gradually prior to ejection failure, probably due to back-leak (Figure 4B).

**FIGURE 5:**
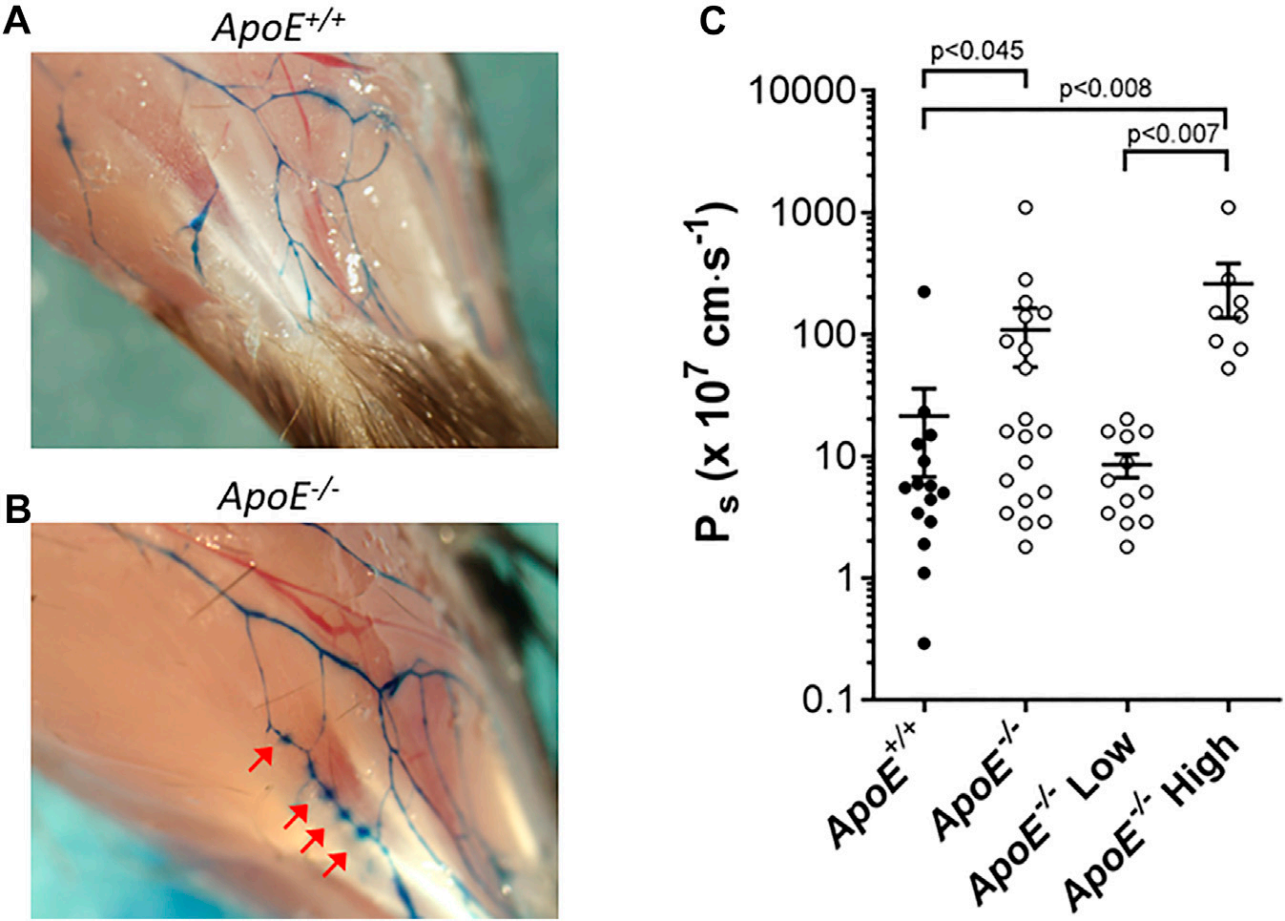
*ApoE* ^*−/−*^ vessels exhibit hyperpermeability. **(A–B)** Increased leak of Evan’s Blue dye is evident *in vivo* in *ApoE* ^*−/−*^ vessels **(B)** compared to *ApoE* ^*+/+*^ vessels **(A)**. Arrows mark sites of leakage. **(C)** *Ex vivo* quantification of albumin permeability (P_s_) for *ApoE* ^*+/+*^ vessels (n = 15) and *ApoE* ^*−/−*^ vessels (n = 20). The averages were 21 × 10^–7^ cm/s vs. 109 × 10^–7^ cm/s for the controls vs. knockouts, respectively, which was significantly different based on a Mann-Whitney test. There was high variability in the *ApoE* ^*−/−*^ group, with 8 of 20 *ApoE* ^*−/−*^ vessels having severely elevated permeability (statistically significant by Mann-Whitney test) compared to *ApoE* ^*+/+*^ vessels when the data were split into subpopulations, and 12 of 20 vessels were within the range of *ApoE* ^*+/+*^ vessels (not significantly different by Mann-Whitney test).

Next, we measured the permeability of collecting lymphatics to albumin. Previous observations in *ApoE* ^*−/−*^ mice showed that Evans Blue, after injection into the interstitium of the ear and uptake into initial lymphatics, appeared to leak from the collecting vessels draining the ear (Lim et al., 2009). We confirmed those results in the hindlimb, as shown in Figures 5A,B. The injection of Evans blue into the foot resulted in its subsequent appearance in the two main popliteal lymphatics in the calf. In *ApoE* ^*+/+*^ mice, the borders of these vessels were sharply defined, whereas in *ApoE* ^*−/−*^ mice focal spots of dye leakage from the popliteal collectors were evident in *ApoE* ^*−/−*^ mice (arrows). The near-uniform spacing suggests that the leakage was possibly occurring at valve sites. Because subsequent analysis of dye leakage would have been highly subjective, we quantified the permeability of popliteal collectors using *ex vivo* methods similar to those previously described in detail (Scallan et al., 2015).

For permeability measurements, a collecting lymphatic was dissected from the mesentery, taking advantage of the lack of strong spontaneous contractions of lymphatics in this tissue bed. After cannulation and cleaning, a vessel was perfused through a ‘theta’ micropipette that contained two identical solutions, one of which contained albumin tagged with a fluorophore. Initially, a washout solution was perfused to obtain a baseline recording on the photometer. Switching to the other half of the micropipette selectively perfused the fluorescent albumin without a change in intraluminal pressure. Over time, fluorescent albumin moved across the lymphatic wall. The photometer voltage increased rapidly after the solution change, followed by a slow increase due to albumin movement across the vessel wall. Permeability (Ps) could be determined from the slope of the steady-state portion of the recording, which was fit using linear regression. Measurement of mesenteric collecting lymphatic Ps revealed a loss of barrier function in *ApoE* ^*−/−*^ vessels (109 × 10^–7^ cm/s; n = 15) compared with *ApoE* ^*+/+*^ controls (21 × 10^–7^ cm/s; n = 20) that was statistically significant (Figure 5C). Further analysis of the data revealed that there were two populations of *ApoE* ^*−/−*^ vessels, one with normal permeabilities (*ApoE* ^*−/−*^ Low) and one with significantly elevated permeabilities (*ApoE* ^*−/−*^ High). The reasons for these differences are not clear but suggest that some *ApoE* ^*−/−*^ vessels may be more susceptible to damage by whatever combination of factors is mediating the hyperpermeability.

**FIGURE 6:**
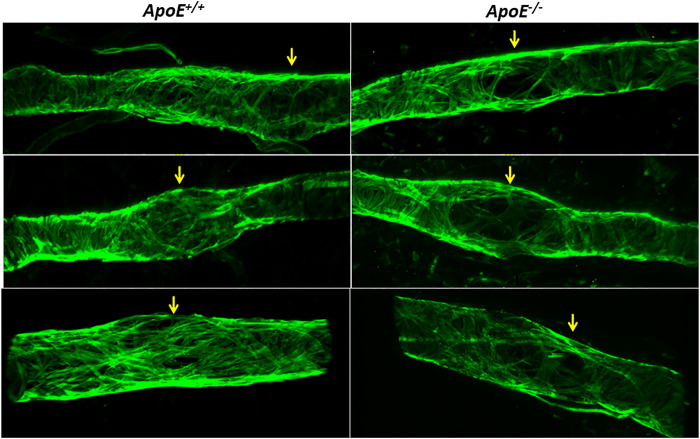
Low Investiture of lymphatic muscle cells at valve areas. Three examples showing that, compared to *ApoE* ^*+/+*^ vessels, *ApoE* ^*−/−*^ vessels have reduced LMC coverage in valve sinus regions. LMCs were stained with anti-SMA. Valve regions were identified by CD31 staining (not shown for clarity).

Subsequent imaging of the valve regions of popliteal collectors *in situ* revealed that many valves in *ApoE* ^*−/−*^ mice also had reduced LMC coverage in their sinus regions. Three examples are shown in Figure 6 along with three examples from *ApoE* ^*+/+*^ vessels, in which the green fluorescence signal denotes SMA staining to mark the locations of LMCs, with unusually large gaps occurring at valve sites; valves at those sites were identified based on CD31 staining (not shown).

## Discussion

Despite existing evidence that hypercholesterolemia impairs lymphatic drainage, it was unknown whether functional alterations in collecting lymphatics contributed to this impairment. Through comprehensive analyses of collecting lymphatic function using vessels from hypercholesterolemic *ApoE* ^*−/−*^ mice, we demonstrate here that hypercholesterolemia significantly compromises the contractile, valve and barrier functions of collecting lymphatics.

Hypercholesterolemia is the major risk factor for atherosclerotic disease and is also a common clinical feature in diabetes, obesity and some autoimmune diseases. Obesity relates to the accumulation of excess body fat, as determined by the measurement of BMI, and is also a major risk factor for cardiovascular diseases. Although obesity is frequently associated with hypercholesterolemia and the combination of both conditions worsens cardiovascular risk status, many obese patients exhibit a normal blood lipid profile (Miettinen, 1971). Likewise, many hypercholesterolemic patients (Guerrero-Romero & Rodriguez-Moran, 2006) as well as *ApoE* ^*−/−*^ mice (Schreyer et al., 2002; Chiba et al., 2003; Hofmann et al., 2008) are not obese. However, obvious similarities between obesity and hypercholesterolemia include the deposition of lipids in extravascular tissues in the form of white adipose expansion or foam cells and the association of lipids with impaired lymphatic drainage (Khan et al., 2022).

A number of studies using mouse models of obesity induced by HFD have reported a clear association between obesity and impaired lymph transport (Weitman et al., 2013; Blum et al., 2014; Savetsky et al., 2014; Nitti et al., 2016). The impairment of lymphatic flow is in part due to vessel wall dilation and in part to reduced contractile activity of collecting lymphatics (Blum et al., 2014). The degree of lymphatic dysfunction appears to be related to the composition of the high fat diet. In obesity induced by feeding mice a Western diet, impairment of lymphatic flow was associated only with collecting lymphatic valve dysfunction and not impaired contraction amplitude (Castorena-Gonzalez, 2022); this is in contrast to obesity induced by HFD, which is characterized by both contractile and (apparent) valve dysfunction (Blum et al., 2014; Nitti et al., 2016). These differences in phenotype may be explained by differences in the percentage of fat present in the high fat and Western diets (60% in high fat diet *versus* 40% in Western diet). Like the effect of adipose tissue expansion on collecting lymphatics, hypercholesterolemia in *ApoE* ^*−/−*^ mice reduced the contractile function of collecting vessels (Figures 1, 4) and compromised valve function. Lymphatic valves in *ApoE* ^*−/−*^ mice were characterized by increased back-leak (Figure 2) and an increase in ΔP for closure (Figure 3), which we infer reflects an increase in leaflet stiffness, as supported by modelling studies (Bertram, 2020); however, actual stiffness measurements (e.g., reflected as a shift in the biaxial stress-strain relationship of the leaflets) are not presently feasible in these small structures. Notably, valve dysfunction in the Western diet-induced obesity model was only observed in mesenteric and not in popliteal collecting lymphatics (Castorena-Gonzalez, 2022), in contrast to the hyperpermeability of mesenteric collectors and both valve and contractile dysfunction in popliteal collectors of *ApoE* ^*−/−*^ mice fed a HFD. Moreover, the levels of valve dysfunction in *ApoE* ^*−/−*^ mice (Figures 2, 3) were greater than those in mice fed only a Western diet [see Figure 2 in (Castorena-Gonzalez, 2022)]. Although the levels of cholesterol increased in Western diet fed mice from 80 mg/dl to 120 mg/dl, these levels remain rather low compared to those in hypercholesterolemic *ApoE* ^*−/−*^ mice which reach 1,000–2000 mg/dl. These comparisons further support the idea that diet composition may differentially affect collecting vessel function and appropriate caution should be used when comparing results from diet-induced obesity and hypercholesterolemic mouse models.

The differences between the phenotypes of mice on a HFD vs. Western diet also raise the issue of the influence of perivascular fat on lymphatic function. Although there were no obvious differences in the amount of fat associated with *ApoE* ^*+/+*^ and *ApoE* ^*−/−*^ popliteal vessels *in vivo,* as indicated by the images in Figure 5A, we cannot state whether there were quantitative differences in vessel-associated fat between the two strains. However, in all of our *ex vivo* functional tests, the fat was removed from the lymphatic vessel in order to allow internal diameter measurement and/or visualization of the valve(s). Thus, any acute effects of perivascular fat were eliminated. Vessels in which all perivascular fat remained intact would not have be amenable to tests of valve function, pump function or permeability. Although studies such as Harvey *et al.* (Harvey et al., 2005) and Cao *et al.* (Cao et al., 2021) have demonstrated an association between fat accumulation and lymphatic collector permeability, to our knowledge, there are no studies looking at the influence of perivascular vessel adipose tissue (PVAT) on lymphatic contractile function or valve function using techniques such as those routinely used in arteries to show the modulatory effects of PVAT (Agabiti-Rosei et al., 2018; Hillock-Watling & Gotlieb, 2022). This remains an area for further investigation.

In the present study, we also detected an elevated permeability of collecting vessels in hypercholesterolemic *ApoE* ^*−/−*^ mice, as previously noted for collecting vessels of diabetic mice (Scallan et al., 2015). Although our analysis revealed that hypercholesterolemia significantly altered the barrier function of collecting lymphatics, hyperpermeability was only characteristic of half the *ApoE* ^*−/−*^ vessels (Figure 5). Although the permeability of this cohort of vessels was elevated 5-fold, that elevation was rather modest compared to the 130-fold increase in permeability measured previously in *db/db* lymphatic vessels (Scallan et al., 2015), and the other half of the *ApoE* ^*−/−*^ vessels had normal permeability to albumin. The reasons why only some vessels were susceptible remain unknown. Lymphatic hyperpermeability has been previously demonstrated in another HFD mouse model (Cao et al., 2021) as well as in *Apelin* ^*−/−*^ mice (Sawane et al., 2013), and in both cases the hyperpermeability was reversed to a large extent by COX2 inhibition, suggesting that activation of COX2 may be a common mechanism underlying elevated lymphatic permeability. Whether COX2 activation underlies the permeability changes of *ApoE* ^*−/−*^ vessels in the present study remains to be determined.

Although further study is needed to elucidate the mechanisms by which hypercholesterolemia impairs collecting lymphatic function, several factors may underlie the altered function of LECs and LMCs in this context. The first possibility is that collecting vessels may accumulate cholesterol, which in turn affects their function, as observed in arteries of *ApoE* ^*−/−*^ mice. This might be addressed by additional analyses of vessels by light and electron microscopy. Like obesity, hypercholesterolemia is associated with increased oxidative stress and the systemic and/or local production of reactive oxygen species (ROS) and nitric oxide (NO). Various ROS molecules, such as H_2_O_2_, are known to impair contractile activity (Zawieja et al., 1991; Zawieja & Davis, 1993), in part through the activation of ATP-sensitive K^+^ (K_ATP_) channels in LMCs (Davis et al., 2022); increased K^+^ efflux through this channel leads to LMC hyperpolarization and the slowing of spontaneous contractions (Davis et al., 2022). It is possible that K_ATP_ channel activation contributes to the lower frequency of spontaneous contraction in *ApoE* ^*−/−*^ vessels (Figure 1E), as was previously shown for mesenteric lymphatic collectors from rats fed a high fructose diet (Zawieja et al., 2016), but that possibility remains to be tested. NO production may also inhibit lymphatic contractions, but the effects of NO on lymphatic contractile function are complex. Low NO concentrations, such as those associated with pulsatile flow from spontaneous contractions, enhance diastolic filling and contraction amplitude (Gasheva et al., 2006), but higher NO concentrations, such as those associated with iNOS activation and cytokine production (Liao et al., 2011; Torrisi et al., 2016) inhibit both contraction frequency and amplitude (Scallan & Davis, 2013; Rehal & von der Weid, 2015; Chen et al., 2017; Kim et al., 2021; Davis et al., 2022). The contributions of ROS and NO to the impaired contractile responses of *ApoE* ^*−/−*^ vessels shown in Figures 1, 4 remain to be determined.

The increased oxidative environment in hypercholesterolemia is also known to participate in the modification of low-density lipoproteins. These modified lipoproteins are then taken up by macrophages through scavenger receptors, which in turn become foam cells. Interestingly, two recent studies revealed the expression of scavenger receptors for modified low-density lipoprotein including MSR-1 (SR-A1), MARCO (SR-A6), and CD36 on lymphatic endothelial cells (Berendam et al., 2019; Fujimoto et al., 2020). This raises the possibility that LEC function, including the regulation of permeability, is altered through the uptake of these modified low-density lipoproteins.

Hypercholesterolemia may also compromise the function of collecting lymphatics by altering their structure. This idea is consistent with our observations of reduced LMC coverage in the valve sinus regions of collecting vessels (Figure 6) as well as relatively high levels of LYVE-1 expression (Lim et al., 2009). LYVE-1 is normally downregulated in LECs of mature collecting lymphatics (compared to lymphatic capillaries), suggesting that either complete vessel maturation is retarded or a reversion to a less differentiated phenotype in *ApoE* ^*−/−*^ collectors is triggered. The loss of SMC coverage in the valve regions may explain some of the contractile amplitude impairment, but that seems unlikely to be the complete explanation as the LMC loss was highly localized. Interestingly, at least two other conditions are also associated with LMC loss from collecting lymphatics and impaired contractions, including overproduction of the cytokine TNFα (Kenney et al., 2022a) and exposure to bacterial toxins (Jones et al., 2018). A common theme underlying LMC loss in both the cases, and possibly in *ApoE* ^*−/−*^ mice on HFD, may be elevated cytokine and/or ROS production (Kenney et al., 2022b). LMC loss at valve sinuses could theoretically impact permeability by disrupting LEC-LMC interactions or by increasing the fragility of the sinuses so that they become more susceptible to pressure overload. Decreased FOXC2 expression in LECs of *ApoE* ^*−/−*^ mice (Lim et al., 2013) may also contribute to the observed valve dysfunction in these mice as FOXC2 deficiency has been shown to compromise valve function by enhancing back-leak and increasing leaflet stiffness, making it more difficult for the valves to close (Sabine et al., 2015; Castorena-Gonzalez et al., 2020). An increase in leaflet stiffness would point to possible changes in the extracellular matrix in the valve leaflets and/or the vessel wall, similar to the increased arterial stiffness known to be associated with collagen accumulation in the remodelled arterial wall (Bakker et al., 2005; Martinez-Lemus et al., 2011). Consistent with this hypothesis, obesity-associated fibrosis has been proposed to contribute to reduced lymph transport in a mouse model of secondary lymphedema (Savetsky et al., 2014).

Given the role of lymphatic vessels in immunity (Oliver et al., 2020), the dysfunction of lymphatics observed in hypercholesterolemic mice is expected to affect the immune response. Indeed, poor lymphatic drainage in these mice may compromise the transport of antigens, trafficking of immune cells, antigen presentation and downstream T-cell activation. This is in line with previous studies from our labs and others revealing impaired dendritic cell migration (Lim et al., 2009; Lim et al., 2013) and T-cell egress *via* lymphatics in *ApoE* ^*−/−*^ mice (Tay et al., 2019) as well as higher susceptibility to bacterial, viral or fungal infections in these mice (Moazed et al., 1997; Netea et al., 1997; de Bont et al., 1999; Ludewig et al., 2001; Martens et al., 2008).

Finally, lymphatic dysfunction in *ApoE* ^*−/−*^ mice may also facilitate the progression of atherosclerosis by interfering with the efficiency of reverse cholesterol transport, an atheroprotective mechanism dependent on lymphatic transport (Lim et al., 2013; Martel et al., 2013). This conclusion is supported by our previous data demonstrating that the ligation of lymphatic vessels draining the aorta promotes the accumulation of cholesterol in the arterial wall (Lim et al., 2013; Martel et al., 2013). Lymphatic drainage may also aid in the clearance of inflammatory mediators that contribute to the progression of atherosclerosis. Moreover, we recently presented evidence that the efficient lymphatic drainage of the aorta is required for the therapeutic effect of the cholesterol-lowering drug, ezetimibe (Yeo et al., 2020). Therefore, targeting lymphatic function would seem to be a promising approach to treat cardiometabolic diseases.

## Data Availability

The raw data supporting the conclusion of this article will be made available by the authors, without undue reservation.
